# Supplementary material for: Viral load-driven systemic immune exhaustion is an enabler of antibody breadth in HIV infection
Source: bioRxiv. 2026 Jun 17:2026.06.05.730519. Preprint. [Version 2] doi: 10.64898/2026.06.05.730519 (PMC13278038; doi:10.64898/2026.06.05.730519)
Supplement: Supplementary file 1 [file NIHPP2026.06.05.730519v2-supplement-10.pdf]

bioRxiv preprint doi: https://doi.org/10.64898/2026.06.05.730519; this version posted June 17, 2026. The copyright holder for this preprint (which was not certified by peer review) is the author/funder, who has granted bioRxiv a license to display the preprint in perpetuity. It is made available under a CC-BY 4.0 International license.

Supplementary Figures

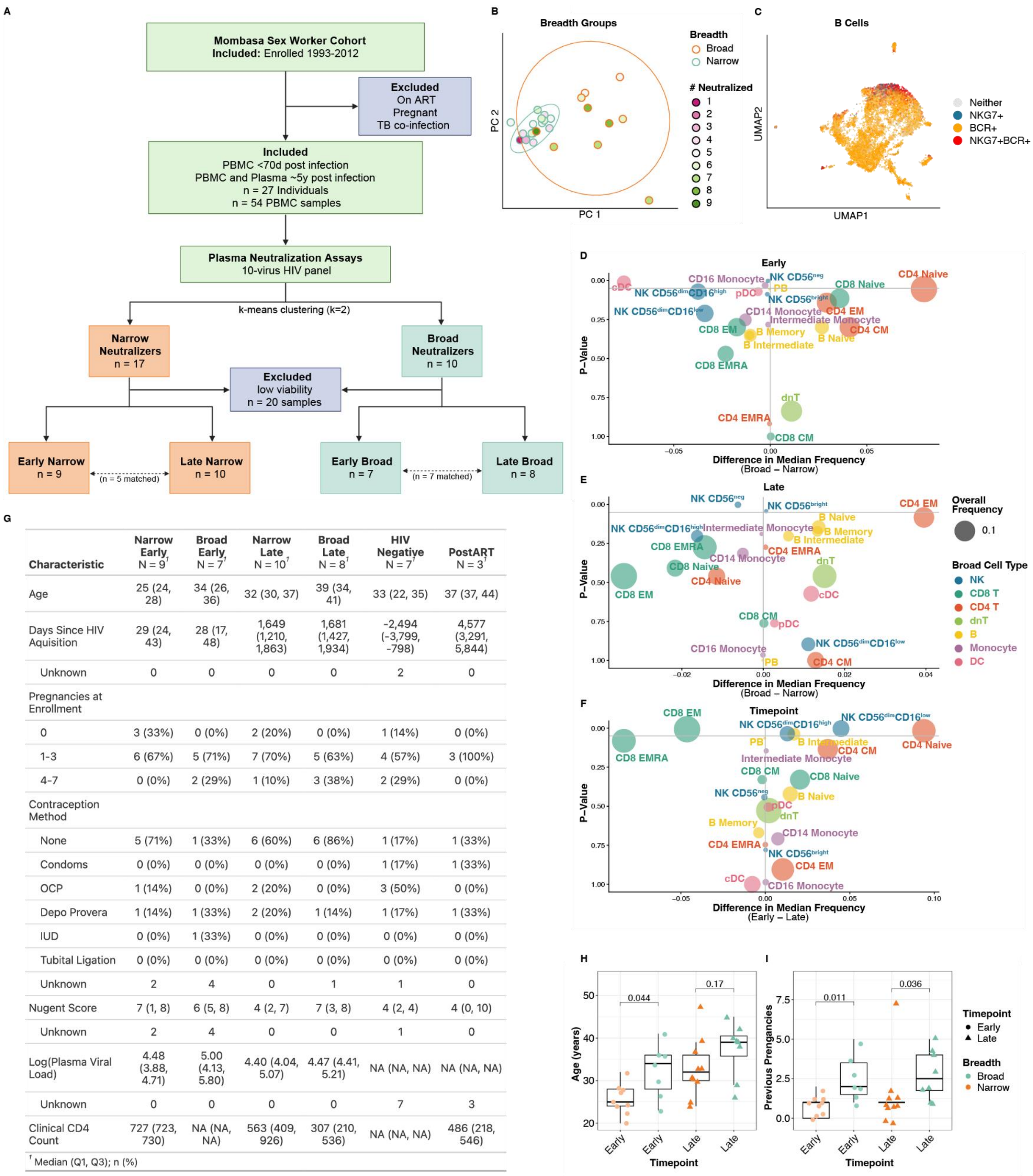

Characteristic	Narrow Early N = 9[1]	Broad Early N = 7[1]	Narrow Late N = 10[1]	Broad Late N = 8[1]	HIV Negative N = 7[1]	PostART N = 3[1]
Age	25 (24, 28)	34 (26, 36)	32 (30, 37)	39 (34, 41)	33 (22, 35)	37 (37, 44)
Days Since HIV Aquisition	29 (24, 43)	28 (17, 48)	1,649 (1,210, 1,863)	1,681 (1,427, 1,934)	-2,494 (-3,799, -798)	4,577 (3,291, 5,844)
Unknown	0	0	0	0	2	0
Pregnancies at Enrollment						
0	3 (33%)	0 (0%)	2 (20%)	0 (0%)	1 (14%)	0 (0%)
1-3	6 (67%)	5 (71%)	7 (70%)	5 (63%)	4 (57%)	3 (100%)
4-7	0 (0%)	2 (29%)	1 (10%)	3 (38%)	2 (29%)	0 (0%)
Contraception Method						
None	5 (71%)	1 (33%)	6 (60%)	6 (86%)	1 (17%)	1 (33%)
Condoms	0 (0%)	0 (0%)	0 (0%)	0 (0%)	1 (17%)	1 (33%)
OCP	1 (14%)	0 (0%)	2 (20%)	0 (0%)	3 (50%)	0 (0%)
Depo Provera	1 (14%)	1 (33%)	2 (20%)	1 (14%)	1 (17%)	1 (33%)
IUD	0 (0%)	1 (33%)	0 (0%)	0 (0%)	0 (0%)	0 (0%)
Tubital Ligation	0 (0%)	0 (0%)	0 (0%)	0 (0%)	0 (0%)	0 (0%)
Unknown	2	4	0	1	1	0
Nugent Score	7 (1, 8)	6 (5, 8)	4 (2, 7)	7 (3, 8)	4 (2, 4)	4 (0, 10)
Unknown	2	4	0	0	1	0
Log(Plasma Viral Load)	4.48 (3.88, 4.71)	5.00 (4.13, 5.80)	4.40 (4.04, 5.07)	4.47 (4.41, 5.21)	NA (NA, NA)	NA (NA, NA)
Unknown	0	0	0	0	7	3
Clinical CD4 Count	727 (723, 730)	NA (NA, NA)	563 (409, 926)	307 (210, 536)	NA (NA, NA)	486 (218, 546)

[1] Median (Q1, Q3); n (%)

Supplementary Fig. 1: Study metadata.
A. Sample outline for study design and data exclusion. **B.** Scatterplot of principal component (PC) 1 and 2 from PCA of NT50 across 10 HIV pseudoviruses. Each point represents one subject colored by the number of viruses neutralized. Outlines represent breadth groups defined by k-means clustering (k = 2). **C.** UMAP of B cells from scRNA-seq dataset

bioRxiv preprint doi: https://doi.org/10.64898/2026.06.05.730519; this version posted June 17, 2026. The copyright holder for this preprint (which was not certified by peer review) is the author/funder, who has granted bioRxiv a license to display the preprint in perpetuity. It is made available under a CC-BY 4.0 International license.

colored by the presence of a successfully sequenced BCR and/or *NKG7* expression above zero. **D-F.** Volcano plot of median difference in fine cell type frequencies (% of PBMC) in early and late infection (**D**), broad and narrow neutralizers in early infection (**E**), and broad and narrow neutralizers in late infection (**F**). Y axis shows P-value from wilcoxon rank sum test. **G.** Clinical and demographic characteristics of study participants by breadth group and infection timepoint. NA represents missing or irrelevant data. **H.** Boxplot of participant age in each breadth group. **I.** Boxplot of pregnancies before enrollment in breadth groups. P-values by two-sided Wilcoxon rank-sum test with Bonferroni's correction for multiple hypothesis testing. Each point represents one donor.

Firth Confounder-Adjusted Regression — STABL Features

M1: +age +pregnancies   M2: +log VL (+CD4 if late)

				M1 adjustment		M2 adjustment	
**Modality**	**Cell type**	**Feature**	**β M0**	**β M1**[1]	**% mod M1**	**β M2**[1,2]	**% mod M2**
**Early**							
Protein	NK	CD38	3.545	2.947	-16.9%	2.471	-30.3%
Protein	CD4T	CD40L	1.885	1.954	3.6%	2.767	46.8%
Protein	CD4T	GRANZYME-B	1.912	1.231	-35.6%	2.367	23.8%
Protein	CD8T	GRANZYME-B	3.180	3.217	1.2%	2.456	-22.8%
RNA	Monocyte	APOBEC3A	1.578	1.717	8.8%	3.330	111.1%
RNA	Monocyte	IFITM3	1.277	2.337	82.9%	2.394	87.4%
RNA	Monocyte	SAT1	1.521	1.905	25.2%	1.925	26.6%
RNA	B	SAT1	1.140	1.691	48.3%	1.367	19.9%
RNA	DC	TYROBP	1.764	2.357	33.6%	2.369	34.3%
BCR	Heavy Chain	IGHV1-58*02	1.093	0.995	-9.0%	0.641	-41.3%
BCR	Heavy Chain	IGHV1-8*03	1.088	0.878	-19.3%	0.647	-40.6%
BCR	Heavy Chain	IGHV4-39*07	-1.361	-1.234	9.3%	-1.274	6.4%
BCR	Heavy Chain	IGHJ6*02	1.244	0.510	-59.0%	0.377	-69.7%
BCR	Light Chain	IGKV2-30*01	1.372	1.332	-2.9%	1.026	-25.2%
BCR	Light Chain	IGLV8-61*01	1.587	1.297	-18.3%	1.520	-4.2%
BCR	Light Chain	IGKJ5*01	2.157	1.862	-13.7%	1.471	-31.8%
Clinical	Clinical and Demographic	Age	1.304	—	—	—	—
Clinical	Clinical and Demographic	Log(Viral Load)	0.622	0.725	16.6%	—	—
Clinical	Clinical and Demographic	Pregnancies at Enrollment	1.999	—	—	—	—
**Late**							
Protein	NK	PERFORIN	-1.548	-1.528	1.3%	-1.314	15.1%
Protein	CD8T	CD16	2.805	2.430	-13.4%	2.182	-22.2%
Protein	Monocyte	CCR7-CD197	-1.615	-1.403	13.1%	-2.147	-32.9%
Protein	Monocyte	CCR6	-1.750	-2.617	-49.5%	-1.689	3.5%
Protein	DC	CXCR3-CD183	-1.592	-2.641	-65.9%	-1.936	-21.6%
Protein	DC	CCR6	-1.775	-1.722	3.0%	-1.283	27.7%
RNA	CD8T	HLA-DRB1	1.760	1.542	-12.4%	1.855	5.4%
RNA	B	CD79A	3.483	7.178	106.1%	3.903	12.0%
RNA	B	NKG7	-3.035	-2.847	6.2%	-2.121	30.1%
BCR	Heavy Chain	IGHV1-46*03	1.338	1.097	-18.0%	0.937	-30.0%
BCR	Heavy Chain	IGHV3-48*01	1.684	1.483	-11.9%	1.269	-24.6%
BCR	Heavy Chain	IGHV3-7*04	1.447	1.254	-13.4%	1.153	-20.3%
BCR	Heavy Chain	IGHD3-10*01	2.263	2.100	-7.2%	1.736	-23.3%
BCR	Light Chain	IGKJ2*02	1.429	1.371	-4.1%	1.038	-27.4%
Clinical	Clinical and Demographic	Log(Viral Load)	0.447	0.411	-7.9%	—	—
Clinical	Clinical and Demographic	Clinical CD4 Count	-1.050	-1.085	-3.3%	—	—
Clinical	Clinical and Demographic	Nugent Score	0.494	0.413	-16.5%	0.021	-95.7%
Clinical	Clinical and Demographic	Pregnancies at Enrollment	0.630	—	—	—	—

[1] Age and pregnancies at enrollment: adjustment-only covariates; M1 and M2 not estimated.

[2] lgpvln and cd4: M2 not estimated (feature cannot be its own covariate).

Supplementary Fig. 2: Confounder-adjusted Firth penalized regression results.

Features selected by Stabl were evaluated for percent moderation after adjusting for age, pregnancies, plasma viral load, and CD4 T cell count.

bioRxiv preprint doi: https://doi.org/10.64898/2026.06.05.730519; this version posted June 17, 2026. The copyright holder for this preprint (which was not certified by peer review) is the author/funder, who has granted bioRxiv a license to display the preprint in perpetuity. It is made available under a CC-BY 4.0 International license.

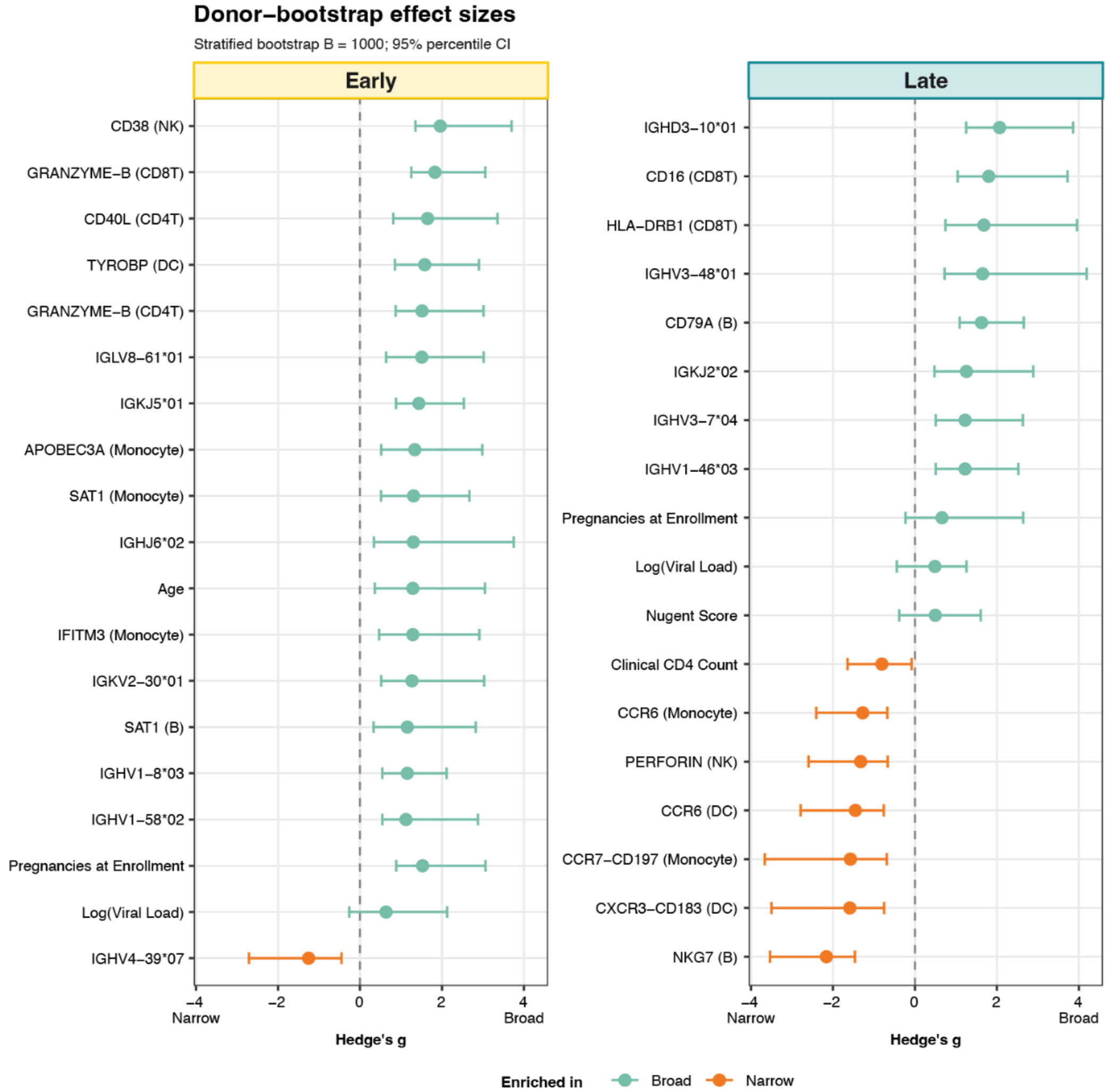

Supplementary Fig. 3: Robustness to donor bootstrapping

Hedge's g was calculated for each Stabl-selected feature in stratified bootstrap resampling (B=1000) groups, independently resampling broad and narrow donors with replacement. Points represent the observed effect size, and error bars estimate the 95% confidence interval (CI).

bioRxiv preprint doi: https://doi.org/10.64898/2026.06.05.730519; this version posted June 17, 2026. The copyright holder for this preprint (which was not certified by peer review) is the author/funder, who has granted bioRxiv a license to display the preprint in perpetuity. It is made available under a CC-BY 4.0 International license.

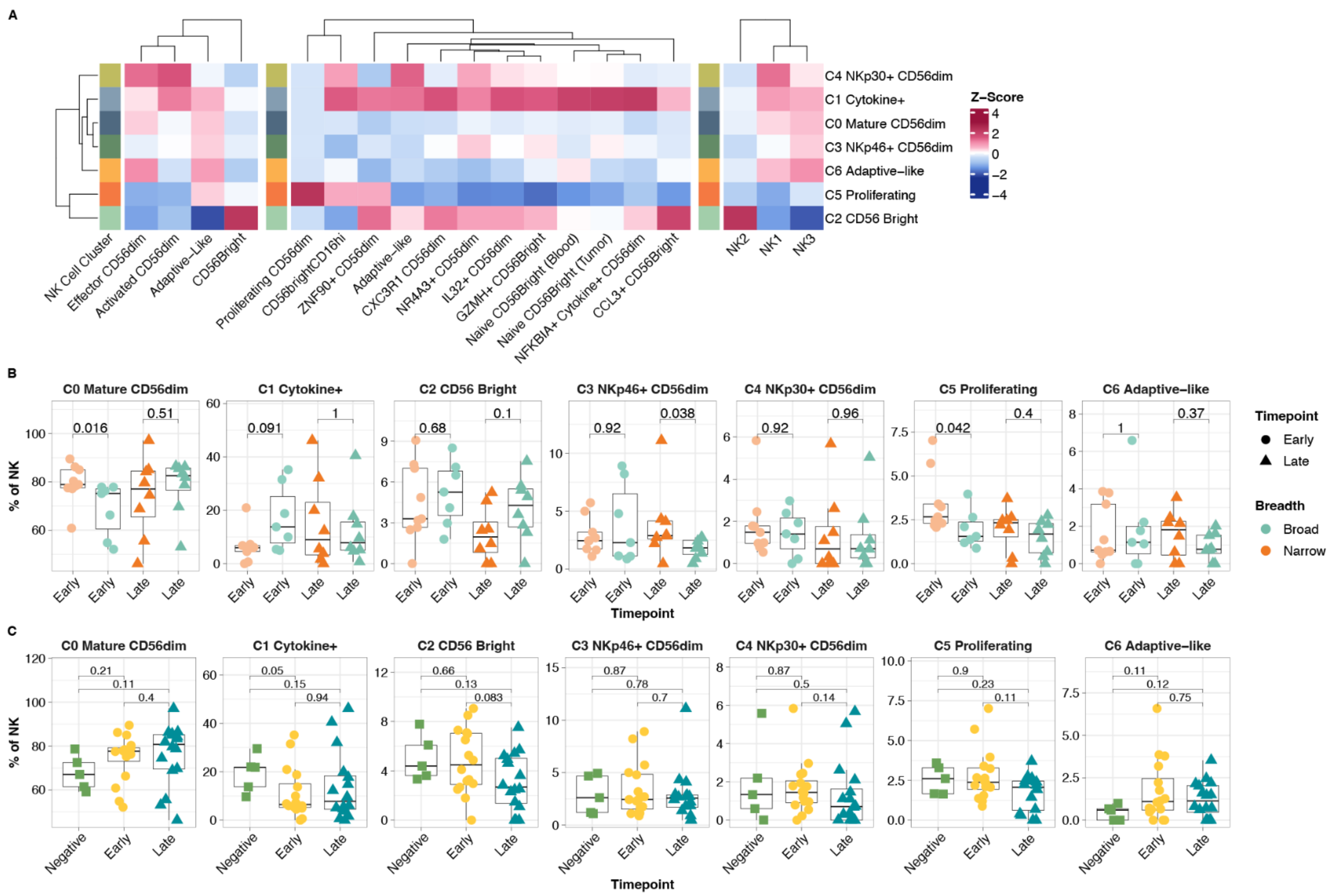

Supplementary Fig. 4: NK cell transcriptomic clusters.
A. Heatmap of normalized expression of NK cell gene signatures from Netskar et al., Tang et al., and Rebuffet et al. in transcriptomic clusters. **B.** Boxplots of cluster frequencies in breadth groups. **C.** Boxplots of cluster frequencies in infection timepoints. P-values by two-sided Wilcoxon rank-sum test with Bonferroni's correction for multiple hypothesis testing. Each point represents one donor.

bioRxiv preprint doi: https://doi.org/10.64898/2026.06.05.730519; this version posted June 17, 2026. The copyright holder for this preprint (which was not certified by peer review) is the author/funder, who has granted bioRxiv a license to display the preprint in perpetuity. It is made available under a CC-BY 4.0 International license.

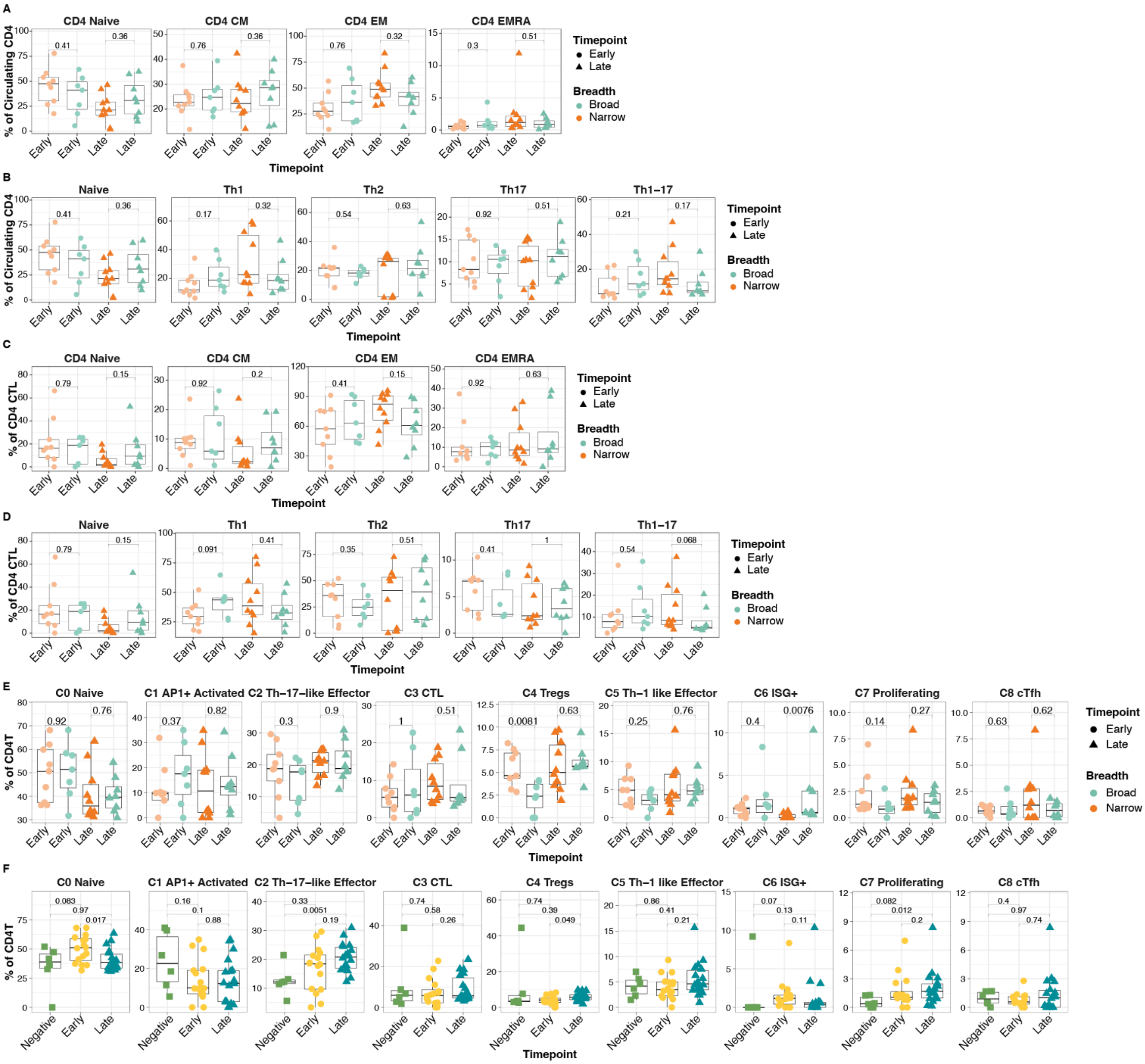

Supplementary Fig. 5: CD4 T cell phenotypes and cell transcriptomic clusters.
A. Boxplots of frequencies of CD4 T memory types between breadth groups. **B.** Boxplots of frequencies of CD4 T helper types between breadth groups. **C.** Boxplots of frequencies of memory types among CD4 CTL between breadth groups. **D.** Boxplots of frequencies of CD4 T helper types among CD4 CTL between breadth groups. **E.** Boxplots of cluster frequencies in breadth groups. **F.** Boxplots of cluster frequencies in infection timepoints. P-values by two-sided Wilcoxon rank-sum test with Bonferroni's correction for multiple hypothesis testing. Each point represents one donor.

bioRxiv preprint doi: https://doi.org/10.64898/2026.06.05.730519; this version posted June 17, 2026. The copyright holder for this preprint (which was not certified by peer review) is the author/funder, who has granted bioRxiv a license to display the preprint in perpetuity. It is made available under a CC-BY 4.0 International license.

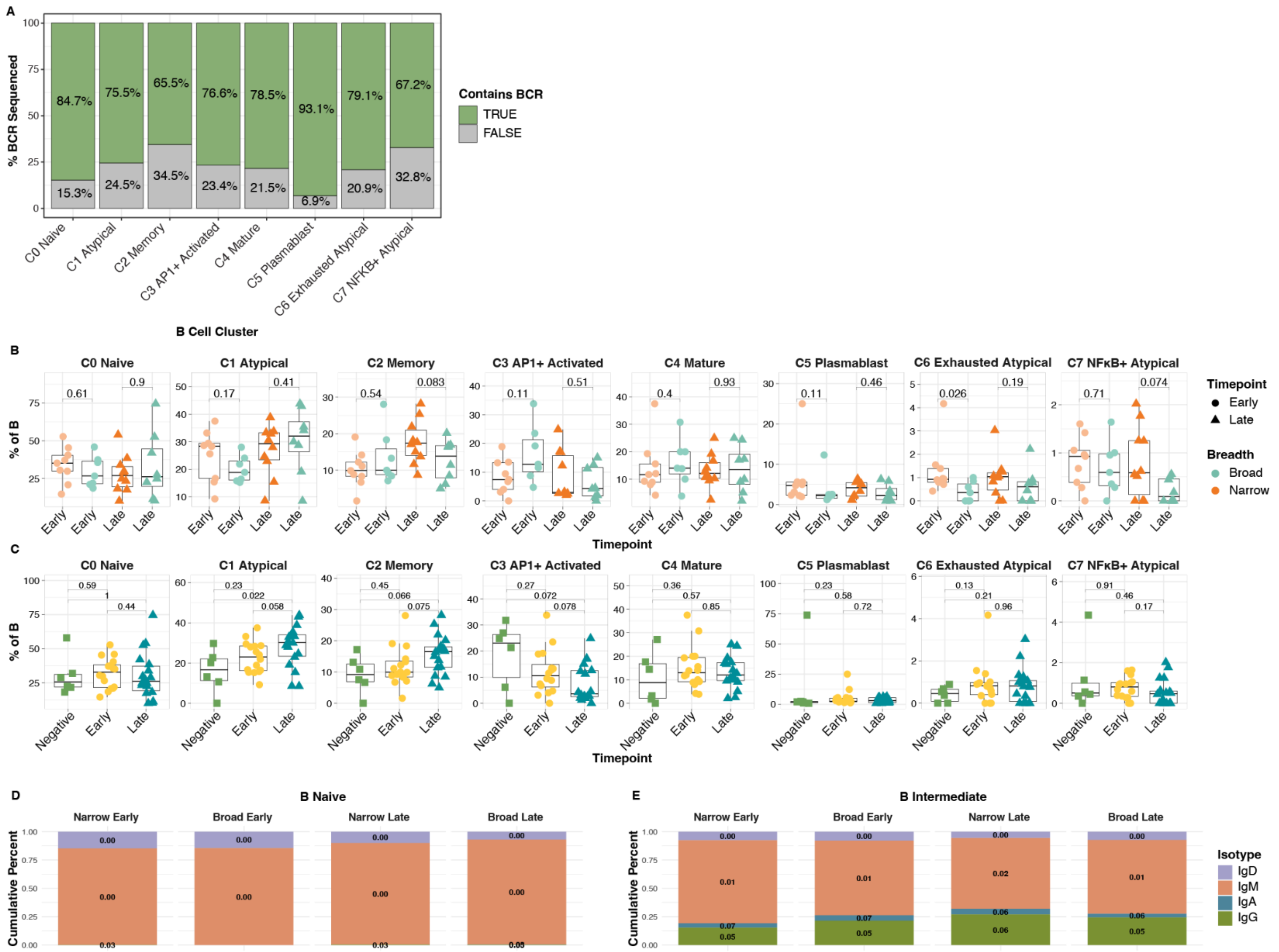

Supplementary Fig. 6: B cell V(D)J sequencing and transcriptomic clusters.

A. Frequency of cells with BCR sequence in transcriptomic clusters. **B**. Boxplots of cluster frequencies in breadth groups. **C.** Boxplots of cluster frequencies in infection timepoints. P-values by two-sided Wilcoxon rank-sum test with Bonferroni's correction for multiple hypothesis testing. Each point represents one donor. **D** and **E.** Cumulative bar plots of immunoglobulin heavy chain gene usage from naive B cells (**D**) and intermediate B cells (**E**) in breadth groups colored by gene with median SHM of each isotype displayed.

bioRxiv preprint doi: https://doi.org/10.64898/2026.06.05.730519; this version posted June 17, 2026. The copyright holder for this preprint (which was not certified by peer review) is the author/funder, who has granted bioRxiv a license to display the preprint in perpetuity. It is made available under a CC-BY 4.0 International license.

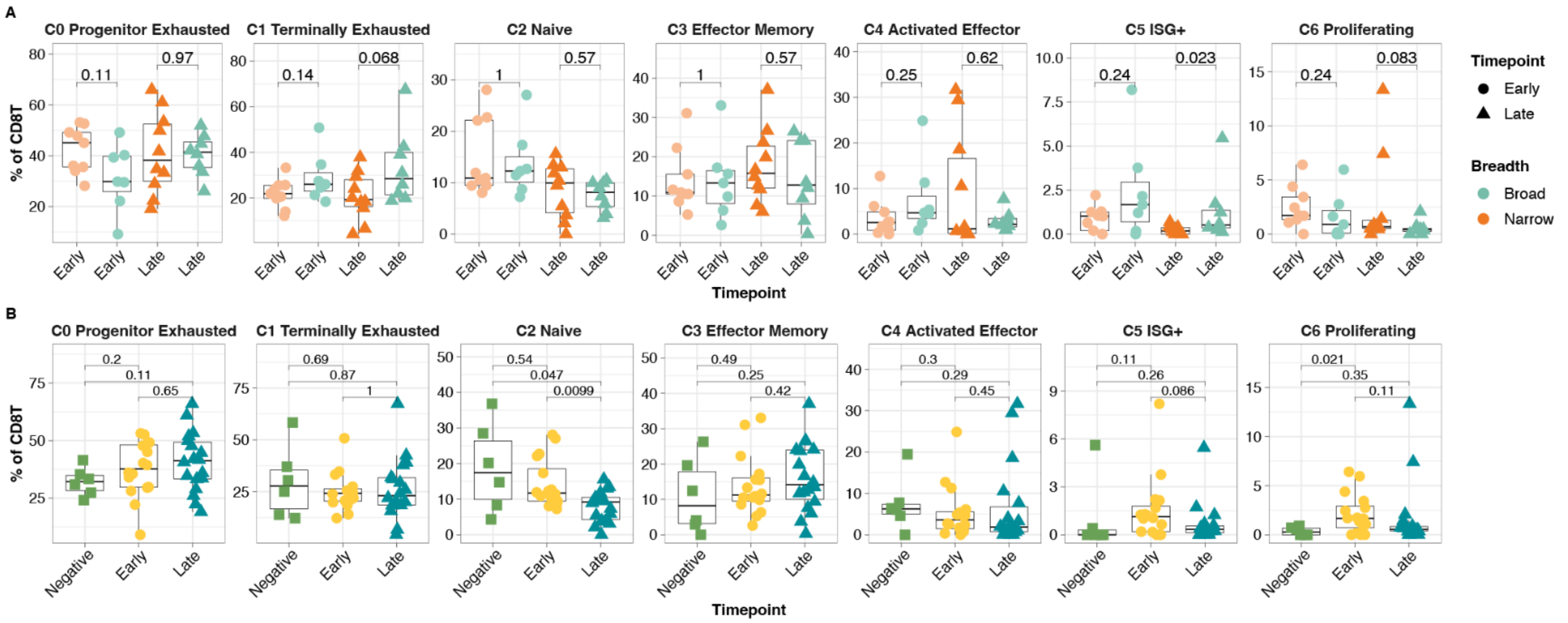

Supplementary Fig. 7: CD8 T cell transcriptomic clusters.

A. Boxplots of cluster frequencies in breadth groups. **B.** Boxplots of cluster frequencies in infection timepoints. P-values by two-sided Wilcoxon rank-sum test with Bonferroni's correction for multiple hypothesis testing. Each point represents one donor.

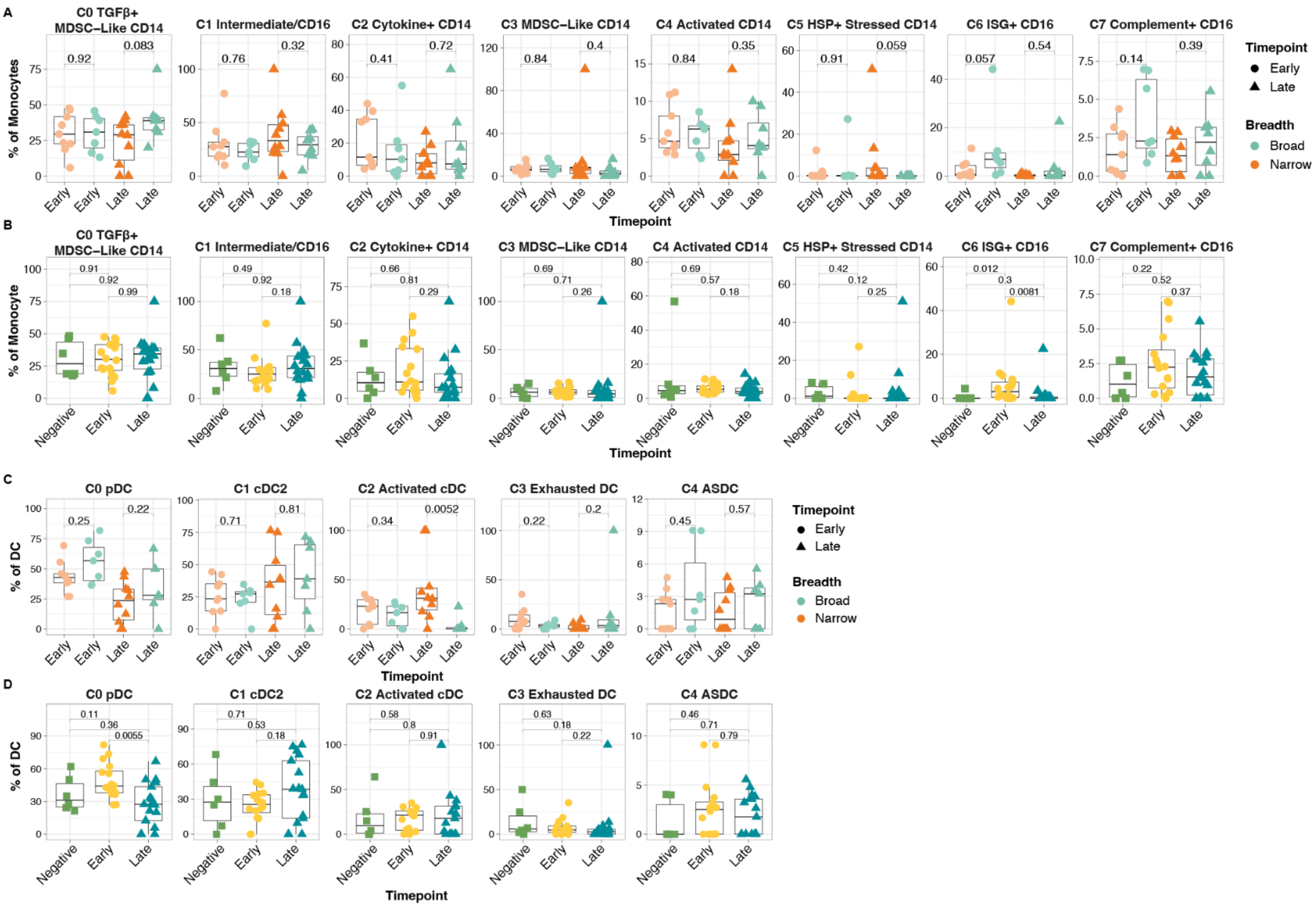

Supplementary Fig. 8: Monocyte and DC transcriptomic clusters.

A. Boxplots of cluster frequencies amongst monocytes in breadth groups. **B.** Boxplots of cluster frequencies amongst monocytes in infection timepoints. **C.** Boxplots of cluster frequencies amongst dendritic cells in breadth groups. **D.** Boxplots of cluster frequencies amongst dendritic cells in infection timepoints. P-values by two-sided Wilcoxon rank-sum test with Bonferroni's correction for multiple hypothesis testing. Each point represents one donor.

bioRxiv preprint doi: https://doi.org/10.64898/2026.06.05.730519; this version posted June 17, 2026. The copyright holder for this preprint (which was not certified by peer review) is the author/funder, who has granted bioRxiv a license to display the preprint in perpetuity. It is made available under a CC-BY 4.0 International license.

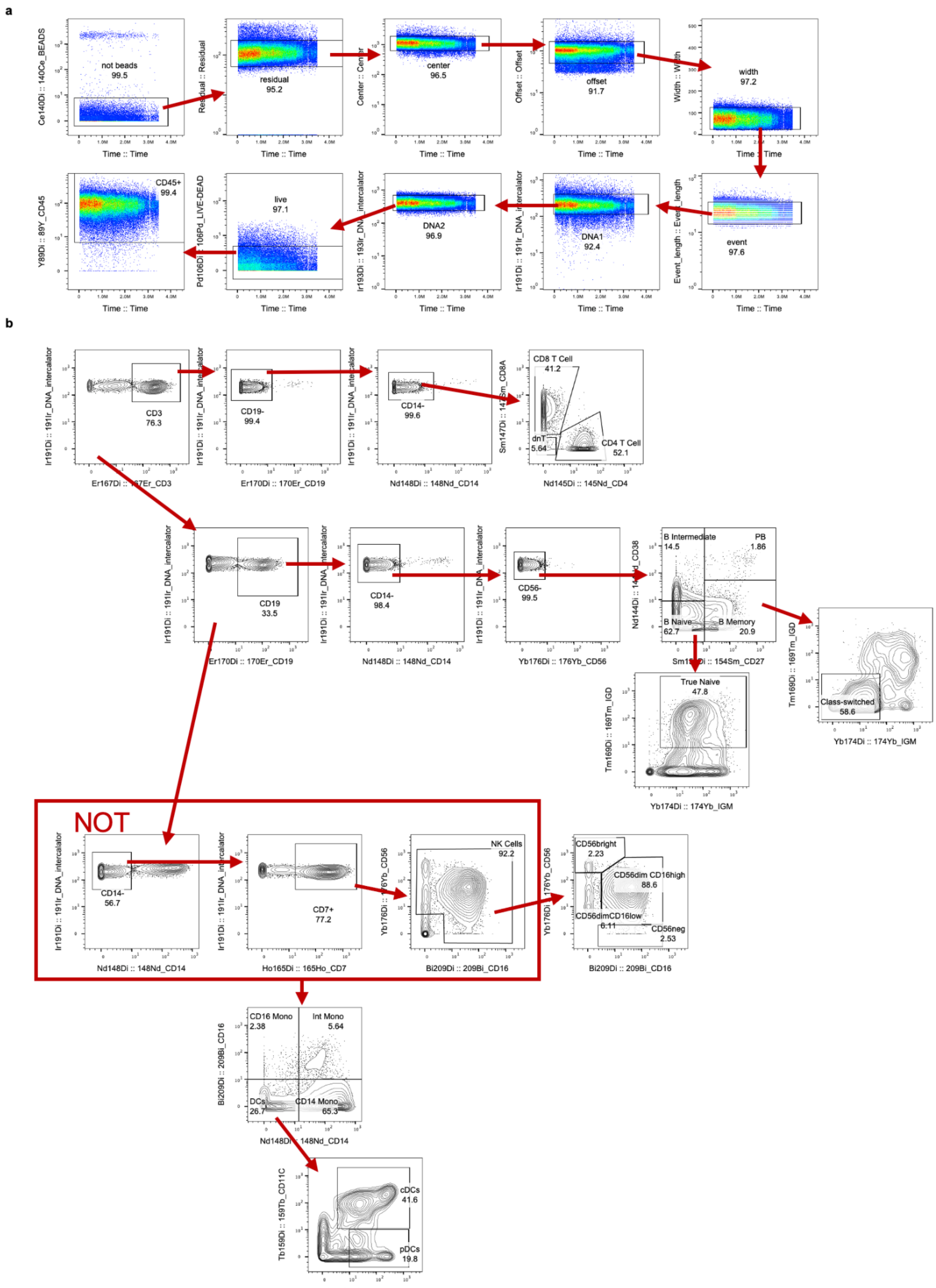

Supplementary Fig. 9: Gating broad cell types in CyTOF data.
A. Representative flow plots of QC gating following Standard Biotools clean up strategy to identify live, CD45+, singlets. **B.** Representative flow plots of gating used to identify CD4 T, CD8 T, NKs and subsets, Monocytes and subsets, DCs and subsets.

bioRxiv preprint doi: https://doi.org/10.64898/2026.06.05.730519; this version posted June 17, 2026. The copyright holder for this preprint (which was not certified by peer review) is the author/funder, who has granted bioRxiv a license to display the preprint in perpetuity. It is made available under a CC-BY 4.0 International license.

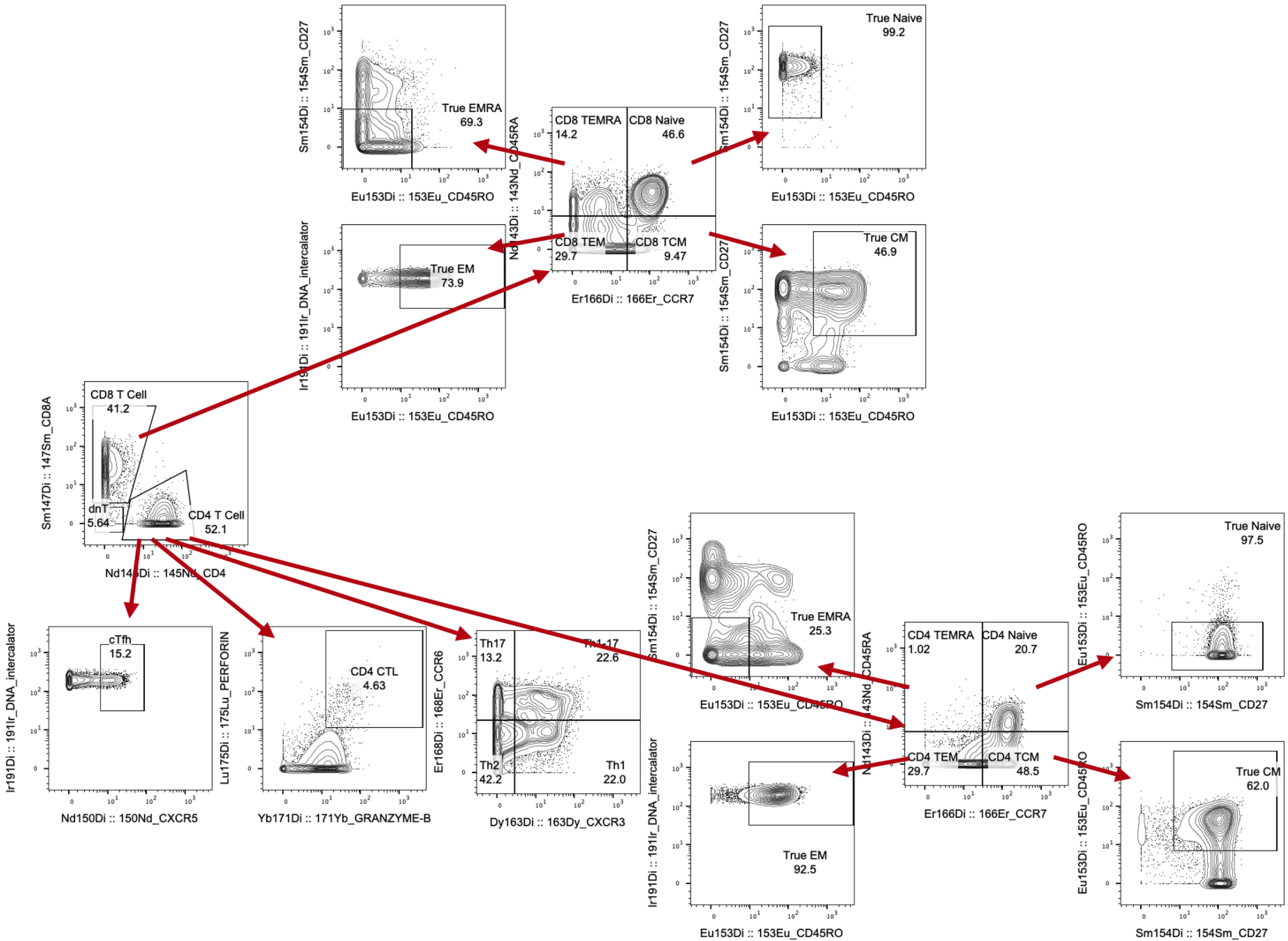

Supplementary Fig. 10 Gating fine T cell subsets in CyTOF data
A. Representative flow plots of gating used to identify CD4 T and CD8 T subsets. For CD4 T cells cTfh status, CTL status, helper subtype, and memory subtype was independently identified for each cell.

bioRxiv preprint doi: https://doi.org/10.64898/2026.06.05.730519; this version posted June 17, 2026. The copyright holder for this preprint (which was not certified by peer review) is the author/funder, who has granted bioRxiv a license to display the preprint in perpetuity. It is made available under a CC-BY 4.0 International license.

Tables:

Table S1: Metadata Deidentified metadata for each sample.

Table S2: Titer Data NT50, PCA, and breadths.

Table S3: Cell Frequencies Differences in cell frequencies between timepoints and breadth groups.

Table S4: Stabl Model Inputs Feature input for each modality.

Table S5: Stabl Model Results Feature selection results for each modality with confounder and donor robustness results.

Table S6 BTMs Blood transcriptional modules plotted for each cell type.

Table S7: Gene Signatures Previously published gene signatures.

Table S8: Multinichenet Top 25 receptor-ligand pairs identified by multinichenet for timepoint and breadth group comparisons.

Table S9: CyTOF Reagents CyTOF antibodies and conjugation kits used.
